# Individual and combined associations of estimated pulse wave velocity and systemic inflammation response index with risk of stroke in middle-aged and older Chinese adults: a prospective cohort study

**DOI:** 10.3389/fcvm.2023.1158098

**Published:** 2023-11-03

**Authors:** Man Xu, Wenqiang Wang, Ruoling Chen, Li Zhou, Hui Hu, Guiyuan Qiao, Ling Wang, Xuezhen Liu, Qiuhong Wang, Yating Ai, Hairong Ren, Ping Hu

**Affiliations:** ^1^ School of Nursing, Hubei University of Chinese Medicine, Wuhan, China; ^2^ Key Laboratory of Environment and Health (HUST), Ministry of Education & Ministry of Environmental Protection, and State Key Laboratory of Environmental Health (Incubation), School of Public Health, Tongji Medical College, Huazhong University of Science and Technology, Wuhan, China; ^3^ Faculty of Education, Health and Wellbeing, University of Wolverhampton, Wolverhampton, United Kingdom; ^4^ Academy of Nutrition and Health, Hubei Province Key Laboratory of Occupational Hazard Identification and Control, School of Public Health, Wuhan University of Science and Technology, Wuhan, China; ^5^ Wuhan Biobank Co., Ltd., Wuhan, China

**Keywords:** systemic inflammation response index, estimated pulse wave velocity, stroke, risk, prospective cohort study

## Abstract

**Background and aims:**

Estimated pulse wave velocity (ePWV) and systemic inflammatory response index (SIRI) have been recently investigated as a marker of arterial stiffness and a novel systemic inflammatory indicator. This study aims to examine the independent and combined association of ePWV and SIRI with incident stroke and its subtypes.

**Methods:**

Data of the Dongfeng-Tongji cohort study was analyzed for 9,154 middle-aged and older adults, who were free of cardiovascular disease and cancer and were followed up to document incident stroke. But their association with incident stroke events and its subtypes have not been well studied. Multivariable adjusted Cox regression models were used to determine the independent and combined association of ePWV and SIRI with incident stroke events.

**Results:**

Over a 7.22-year follow-up, the cohort documented 491 stroke cases (387 ischemic stroke and 104 hemorrhagic stroke). The multivariate adjusted model showed that with each one-unit increase in the level of ePWV, the corresponding hazard ratios (HRs) (95% CI) for total stroke, ischemic stroke, and hemorrhagic stroke were 1.53 (95% CI, 1.23–1.90), 1.42 (95% CI, 1.11–1.83), and 1.92 (95% CI, 1.21–3.03), respectively. Similarly, with each one-unit increase in log-transformed levels of SIRI, the corresponding HRs (95% CI) for total stroke, ischemic stroke, and hemorrhagic stroke were 1.23 (95% CI,1.04–1.47), 1.16 (95% CI, 0.96–1.41), and 1.52 (95% CI, 1.05–2.20), respectively. There appeared to be a combined effect of ePWV and SIRI on stroke; Participants with high levels of both ePWV and SIRI had a higher risk of total stroke and hemorrhagic stroke, with multiple adjusted HR of 2.43 (95% CI, 1.09–5.42). Additionally, the incorporation of ePWV in addition to traditional cardiovascular risk factors significantly improved the predictive accuracy for total stroke with C statistic increased from 0.684 (95% CI, 0.661–0.707) to 0.687 (95% CI, 0.664–0.710; *x*^2 ^= 6.65; *p* for difference = 0.010), and (suggestively) for ischemic stroke with C statistic increased from 0.684 (95% CI, 0.659–0.71) to 0.691(95% CI, 0.666–0.717; *x*^2 ^= 3.13, *p* for difference = 0.077), respectively.

**Conclusions:**

The presence of both high ePWV and SIRI individually, as well as together, was found to be associated with an increased incidence of stroke. The combined stroke risk assessment using these two indicators could potentially improve non-invasive assessment and treatment strategies for high-risk patients, as these indicators are easily accessible in clinical practice.

## 1. Introduction

Stroke is a significant contributor to the global burden of cardiovascular disease (CVD) (1) and is ranked as the second leading cause of disability-adjusted life-years (DALYs) among middle-aged and elderly individuals (2).

Arterial stiffness, that reflects arterial elasticity and function, is often evaluated by measuring the speed at which pulse waves propagate through a section of the vascular system (3). This speed is known as pulse wave velocity (PWV) (3). Studies have shown that elevated levels of PWV are linked to a higher risk of stroke (4). The carotid-femoral pulse wave velocity (cfPWV) is the most widely accepted method for clinically assessing arterial stiffness (3). However, measuring cfPWV requires specialized equipment and trained staff, making it less accessible in clinical practice. In recent years, the Reference Values for Arterial Stiffness Collaboration has developed an equation called estimated pulse wave velocity (ePWV) that estimates aortic stiffness based on age and blood pressure (BP) (5).The ePWV has demonstrated comparable predictive efficiency to cfPWV in predicting major cardiovascular events (6, 7) and Korean Population (8).Several studies have shown the associations of ePWV with cardiovascular outcomes, including stroke events, among Western populations (7, 9). However, there is a lack of research on the relationship between ePWV and cardiovascular outcomes in Chinese populations. Stroke is the leading cause of disability-adjusted life-years (DALY) in China, with a recent report from the China Stroke Statistics Writing Committee indicating that there are 3.94 million new stroke cases and 2.19 million stroke-related deaths annually (2). Moreover, there are notable differences between Chinese and Western populations in terms of overall stroke incidence, proportion of hemorrhagic stroke, and risk factor profiles for intracerebral hemorrhage and ischemic stroke (10, 11). In addition, few studies have examined the associations between ePWV and stroke subtypes. Only one cohort study has investigated the association of ePWV with stroke subtypes in middle-aged men in Finland (12). Since stroke is a heterogeneous disease with diverse pathophysiologies for each subtype, it is crucial to evaluate the relationship between ePWV and each stroke subtype in a larger population. This will help clarify the different pathogenesis of stroke and provide specific insights for effective stroke prevention (13).

Previous population-based studies (14, 15) have also demonstrated an association between arterial stiffness and changes in arterial stiffness with inflammation. Inflammation and immune response are recognized as pivotal factors in the development and activation of atherosclerotic plaques (16). Systemic inflammation refers to a widespread or whole-body inflammatory response, characterized by the release of inflammatory mediators such as cytokines and immune cells, which can occur in various conditions including infections and autoimmune diseases, and circulate throughout the body (17). Systemic inflammatory indexes, such as monocyte to lymphocyte ratio (MLR) and neutrophil to lymphocyte ratio (NLR), have been generated and reported to be related to the prognostic outcome in stroke patients (18–20). Recently, a novel measurement called systemic inflammatory response index (SIRI) has been developed (21). SIRI is based on the counts of peripheral neutrophils, monocytes, and lymphocytes and can reflect the status of local immune response and systemic inflammation in pancreatic cancer patients (21). It has also been identified as an independent prognostic factor for survival in various types of carcinomas (21–23). Additionally, it has shown promise as a low-grade inflammatory indicator for predicting stroke prognosis (24). However, there is limited research on the association between SIRI and incident stroke events due to a lack of available data. Only one study conducted on the Chinese Kailuan cohort showed that elevated SIRI was associated with an increased risk of stroke (25). Additionally, no study has examined the combined effect of SIRI and ePWV on the incidence of stroke.

Therefore, understanding the individual and combined associations of ePWV and SIRI with the risk of stroke in China is crucial for stroke prevention. This paper aims to analyze the data from a large population-based cohort study in China to investigate the individual and combined effects of ePWV and SIRI on incident stroke events.

## 2. Materials and methods

### 2.1. Study population

The participants in this study were selected from the Dongfeng-Tongji cohort study, which was previously described in detail (26). Briefly, from September 2008 to June 2009, a total of 27,009 retirees from Dongfeng Motor Corporation agreed to take part in the survey (26). The aim of the survey was to investigate the associations between lifestyles, occupational and environmental factors, and the risk of chronic diseases (26). Trained interviewers conducted face-to-face interviews with the participants, using a questionnaire to collect information on socio-demographic characteristics, lifestyle, family medical history, history of chronic diseases, and medication history. All participants who completed the baseline investigation were followed until the occurrence of stroke events, death, or December 31, 2016 (the end of the study period), whichever came first.

**Figure 1:**
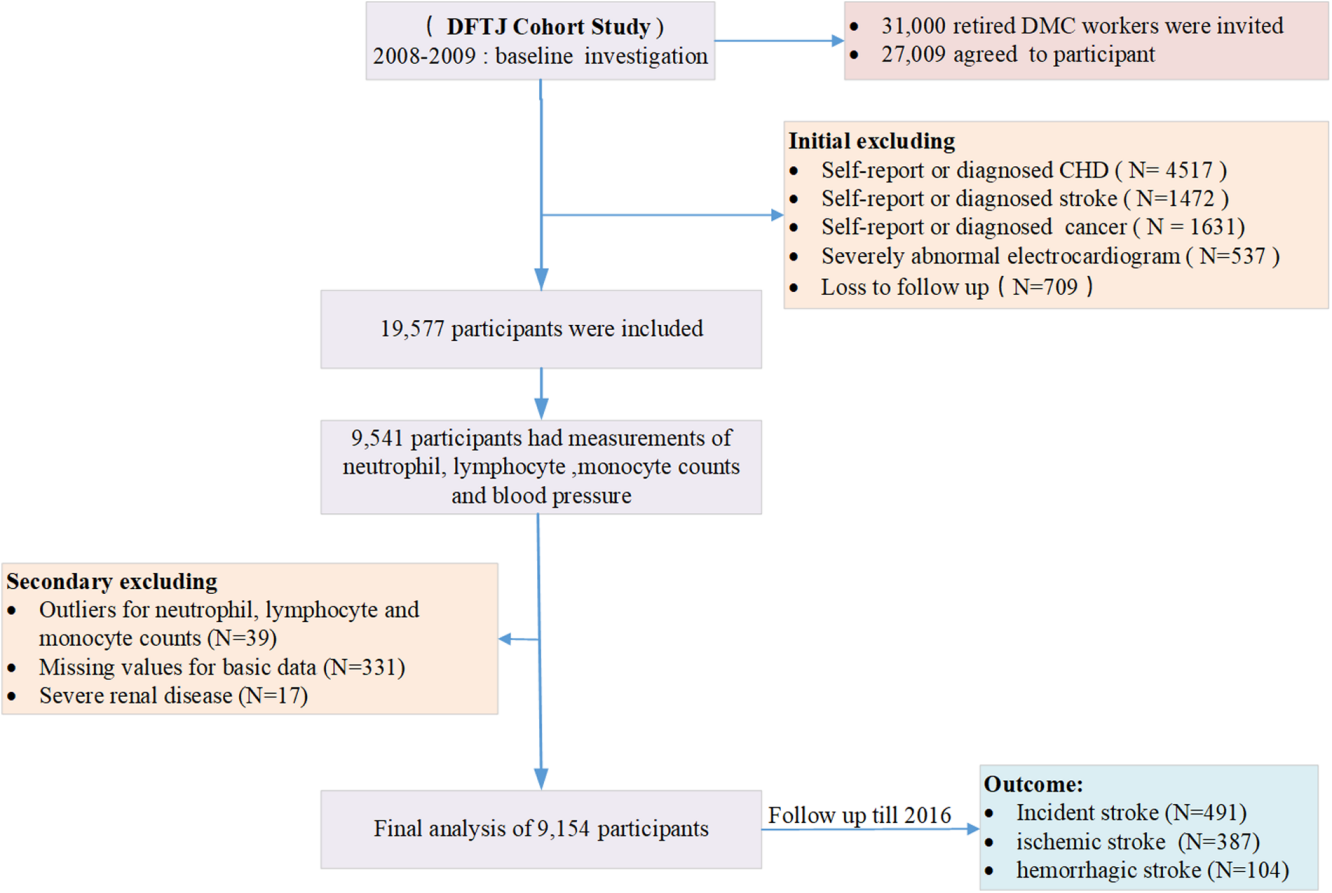
The flow diagram of participants for analysis in our study.

In this study, participants with prevalent self-report cancer, stroke, coronary heart disease (CHD), and severely abnormal electrocardiogram at baseline were excluded. This included conditions such as atrial fibrillation/flutter, complete left bundle branch block, ventricular pre-excitation, atrial tachycardia, premature ventricular contractions, and II or III atrioventricular block (27). Out of the initial 19,557 participants, measurements of lymphocyte, monocyte, and neutrophil counts, as well as BP, were only available for 9,541 participants. Furthermore, we also excluded participants with outliers in their lymphocyte, monocyte, or neutrophil counts, as well as those with inflammatory markers exceeding the general population distribution based on C-reactive protein (CPR) levels (28). Participants with severe renal disease [estimated glomerular filtration rate < 15 ml/min/1.73m^2^, calculated using the equation of Chronic Kidney Disease Epidemiology Collaboration (29)] were also excluded, as their neutrophil counts might be difficult to interpret. Moreover, individuals with missing baseline basic data of body mass index (BMI), smoking status, education levels, and family history of stroke were excluded from the study. The analysis was performed among 9,154 participants, as shown in the flow chart of participant selection process (Figure 1). In comparison to the initial 19,557 participants who were followed up for incident stroke events, participants included in this study were older and had a different distribution of education levels. They also had lower proportions of females and lower levels of body mass index, systolic blood pressure (SBP), triglycerides, LDL-cholesterol, lymphocyte counts, and neutrophil counts. Additionally, they had a lower prevalence of family history of stroke and a higher prevalence of lipid-lowering drugs and aspirins (Supplementary Table S1). Overall, the analytical sample consisted of relatively “healthy” individuals. All authors declare that all supporting data are available within this article. The research protocol of the DFTJ cohort received approval from the Ethics and Human Subject Committee of Tongji Medical College. This study strictly followed the ethical guidelines outlined in the 1975 Declaration of Helsinki. Prior to the implementation of the study, informed consent was obtained from all participants.

### 2.2. Biochemical analyses

The blood cell test, which included peripheral counts of lymphocytes, neutrophils, and monocytes, was conducted within 24 h of venipathic collection of whole blood samples. An automated analyzer, CELL-DYN 3700 (Abbott Laboratories, Abbott Park, Illinois, USA), was used at a clinical laboratory. Additionally, fasting plasma glucose and blood lipids were measured as previously described (30).

### 2.3. Assessment of estimated pulse wave velocity (ePWV)

Using the equation derived by the Reference Values for Arterial Stiffness Collaboration (5), ePWV was calculated from age and MAP. MAP was calculated as MBP = DBP + 0.4 × SBP-DBP (5). The Normal Population equation was used for healthy patients without cardiovascular risk factors, while the equation derived from the Reference Population was used for the other groups since they had at least one cardiovascular risk factor (5).

### 2.4. Assessment of the systemic inflammation response index (SIRI)

The systemic inflammation response index (SIRI) was calculated based on absolute neutrophil count (*N*; × 10^9^/l), monocyte counts (*M*; × 10^9^/l) and lymphocyte counts (*L*; × 10^9^/l) using the formula: SIRI = (*N* × M)/L (21). We transformed SIRI value into a logarithmic scale due to its underlying skewed distribution.

### 2.5. Outcome definition

The outcome of this study focused on the incidence of stroke among participants in our cohort. To track this, we utilized the participants' unique medical insurance card number and ID, which allowed us to link their electronic medical records in Dongfeng Central Hospital with the medical insurance system of Dongfeng Motor Corporation (DMC) (26). As the DMC medical insurance system covers the primary care cost of DMC retirees, we were able to easily connect the electronic medical records of outpatients and inpatients in Dongfeng Central Hospital to the existing database collecting information for the cohort study using the ID and unique medical insurance card number of each subject (26).

Stroke events were identified by inspecting the medical insurance system and medical records at Dongfeng Central Hospital. Events were included if the patient was admitted to the hospital or died from a stroke. A stroke event was defined as the sudden or rapid onset of a typical neurological deficit of vascular origin, lasting for more than 24 h or resulting in death. Stroke diagnosis was confirmed by a panel of physicians based on computed tomography and/or magnetic resonance imaging, as well as the patients' clinical symptoms. Moreover, the total stroke was classified into two categories: ischemic stroke and hemorrhagic stroke. Ischemic stroke was determined based on the criteria of the Trial of Org 10,172 in Acute Stroke Treatment classification, which included large atherosclerosis, small vessel occlusion, cardioembolic infarction, and other subtypes with determined or undetermined causes of infarction (31). Hemorrhagic stroke encompassed both intracerebral hemorrhage and subarachnoid hemorrhage.

### 2.6. Covariates

Smoking status was categorized into current smoker, ex-smoker and nonsmoker. Similarly, dinking status was categorized into current drinker, ex-drinker and nondrinker. Physical activity was defined as regular exercise for more than 20 min each day in the past six months. Office BP was measured in a sitting position after a 5-min rest with the arm at heart level using a mercury sphygmomanometer. Participants were asked if they had used any of the following drugs in the two weeks prior to the collection of their blood sample: anti-hypertensives, lipid-lowering drugs, hypoglycemic agents, insulin, antibiotics, aspirins, thrombolytic agents, and anticoagulant drugs. Drug use for thrombus treatment included the usage of aspirins, thrombolytic agents, and aticoagulant drugs. Participants with a fasting glucose level above 7.0 mmol/L, self-reported doctor-diagnosed diabetes mellitus (DM), or treatment with hypoglycemic agents or insulin were categorized as having prevalent DM.

### 2.7. Statistical analysis

This study involved 9,154 participants. We categorized them based on their baseline ePWV and SIRI quartiles. We described the distributions of participants' baseline characteristics for each category and compared the differences between the four groups. We used Pearson's *x*^2^ tests for categorical variables and ANOVA and Kruskal-Wallis Test for normally and non-normally distributed continuous variables. For each participant, survival time was defined from date of baseline investigation until date of first event, death or date of December 31, 2016.

In the age-sex-adjusted and multivariable Cox proportional hazards regression model, we first calculated hazard ratios (HRs) and confidence interval (95% CI) for incident stroke events by four quartiles of ePWV and SIRI, respectively. Restricted cubic spline was used to examine the potential dose-response relationship and non-linearity association between ePWV or log-transformed SIRI and stroke events (32). Second, according to the median of ePWV levels, participants were categorized into high and low ePWV groups. Similarly, participants were divided into high and low SIRI groups based on the median of SIRI levels. In order to examine the combined association of ePWV and SIRI with stroke, we compared the differences in HRs among four groups: (1) individuals with low levels of both ePWV and SIRI, (2) individuals with high SIRI and low ePWV, (3) individuals with low SIRI and high ePWV, and (4) individuals with high levels of both ePWV and SIRI. To assess the interactive effect of ePWV and SIRI on incident stroke, we tested the statistical significance of ePWV × SIRI in multivariable Cox regression models. Schoenfeld residual plots were plotted to assess whether the assumption of proportional hazards was met. Third, Kaplan–Meier cumulative incidence curves for different groups were plotted and were compared using the log-rank statistic in the unadjusted time-to-stroke event analysis. In addition, to determine if ePWV and SIRI, individually or together add predictive value above the traditional cardiovascular risk factors, model discrimination was assessed with the C statistic by calculating the Uno's concordance statistic from the Cox proportional hazards regression models with different variables.

Referring to the Framingham Stroke Risk Score (33) and the stroke risk prediction model of the China-PAR Project (34), and taking into account the established traditional cardiovascular risk factors, our stroke risk prediction model incorporates age, current smoking status, BMI, SBP, anti-hypertensive drugs, TC, HDL-C, DM, and family history of stroke.

In order to ensure the robustness of our findings, we conducted sensitivity analyses. Firstly, we excluded participants who experienced stroke events within the first two years of follow-up to eliminate potential confounding factors introduced by undiagnosed or pre-symptomatic strokes, as well as pre-existing diseases or health conditions. Secondly, we also considered the impact of chronic respiratory inflammation on our results by making additional adjustments for chronic bronchitis, asthma, and tuberculosis in our main models.

The two-tailed *p*-value <0.05 was considered statistically significant. Data were mainly performed using the SAS software (version 6.4) and the R software (version 3.6.0).

## 3. Results

### 3.1. Characteristics of the cohort participants

Of 9,154 participants, the average age was 63.03 years, and 53.91% were women. Over an average of 7.22 years with a maximum 8.98 years of follow-up, 491 participants developed the first-ever stroke, of which 387 were ischemic stroke and 104 hemorrhagic stroke.

Table 1 presents the baseline characteristics categorized into four quartiles of ePWV and SIRI. There were significant differences observed in age, sex, educational level, BMI, physical activity, drinking status, smoking status, SBP, DBP, fasting blood glucose, triglycerides (TG), total cholesterol (TC), low-density lipoprotein-cholesterol (LDL-C), high-density lipoprotein-cholesterol (HDL-C), past medical history of DM, medication histories (including anti-hypertensive drugs, lipid-lowering drugs, hypoglycemic agents, anticoagulant drugs, aspirins, and antibiotics), and family history of stroke across the four quartiles of ePWV (*p* < 0.05).

**Table 1:** Baseline characteristics of participants by quartiles of ePWV and quartiles of SIRI (*N* = 9,154).

Variable	ePWV				*p*-value	SIRI				*p*-value
	Q1	Q2	Q3	Q4		Q1	Q2	Q3	Q4	
	≤8.84	8.84–9.83	9.83–10.96	≥10.96		<0.48	0.48–0.68	0.68–0.99	≥0.99	
Participants (*N*)	2,306	2,276	2,288	2,284		2,289	2,288	2,288	2,289	
Age, years, mean ± SD^a^	54.91 (5.03)	60.90 (4.04)	64.87 (4.35)	71.50 (5.64)	<0.001	61.84 (7.46)	62.54 (7.70)	63.14 (7.72)	64.60 (7.74)	<0.001
Female, *N* (%)	1,799 (78.0)	1,268 (55.7)	986 (43.1)	882 (38.6)	<0.001	1,649 (72.0)	1,391 (60.8)	1,096 (47.9)	799 (34.9)	<0.001
Education, *N* (%)					<0.001					0.038
<6 years	381 (16.5)	659 (29.0)	729 (31.9)	803 (35.2)		597 (26.1)	628 (27.4)	655 (28.6)	692 (30.2)	
6–8years	882 (38.2)	869 (38.2)	836 (36.5)	708 (31.0)		855 (37.4)	819 (35.8)	810 (35.4)	811 (35.4)	
9–11 years	821 (35.6)	527 (23.2)	457 (20.0)	421 (18.4)		570 (24.9)	593 (25.9)	554 (24.2)	509 (22.2)	
≥ 12 years	222 (9.6)	221 (9.7)	266 (11.6)	352 (15.4)		267 (11.7)	248 (10.8)	269 (11.8)	277 (12.1)	
Body mass index, kg/m^2^^b^	23.37 (3.09)	24.18 (3.16)	24.68 (3.31)	24.70 (3.47)	<0.001	23.66 (3.27)	24.29 (3.30)	24.44 (3.28)	24.54 (3.31)	<0.001
Physical activity, *N* (%)	2,016 (87.4)	2,058 (90.4)	2,086 (91.2)	2,096 (91.8)	<0.001	2,052 (89.6)	2,083 (91.0)	2,075 (90.7)	2,046 (89.4)	0.175
Smoking status, *N* (%)					<0.001					<0.001
Current smokers	335 (14.5)	473 (20.8)	506 (22.1)	405 (17.7)		224 (9.8)	351 (15.3)	511 (22.3)	633 (27.7)	
Ex-smokers	68 (2.9)	184 (8.1)	312 (13.6)	382 (16.7)		159 (6.9)	196 (8.6)	265 (11.6)	326 (14.2)	
Nonsmokers	1,903 (82.5)	1,619 (71.1)	1,470 (64.2)	1,497 (65.5)		1,906 (83.3)	1,741 (76.1)	1,512 (66.1)	1,330 (58.1)	
Drinking status, *N* (%)					<0.001					<0.001
Current drinkers	393 (17.0)	520 (22.8)	575 (25.1)	532 (23.3)		386 (16.9)	469 (20.5)	553 (24.2)	612 (26.7)	
Ex-drinkers	58 (2.5)	114 (5.0)	133 (5.8)	131 (5.7)		63 (2.8)	83 (3.6)	134 (5.9)	156 (6.8)	
Nondrinkers	1,855 (80.4)	1,642 (72.1)	1,580 (69.1)	1,621 (71.0)		1,840 (80.4)	1,736 (75.9)	1,601 (70.0)	1,521 (66.4)	
Systolic blood pressure, mm Hg^a^	111.03 (10.99)	122.35 (11.74)	130.91 (13.56)	142.12 (18.14)	<0.001	123.87 (17.80)	125.82 (17.56)	127.54 (17.78)	129.04 (18.33)	<0.001
Diastolic blood pressure, mm Hg^a^	69.00 (7.29)	73.35 (7.93)	76.46 (9.23)	79.21 (11.51)	<0.001	73.47 (9.54)	74.43 (9.82)	75.08 (9.91)	75.00 (10.20)	<0.001
Triglycerides, mmol/L^b^	1.06 (0.75, 1.53)	1.12 (0.79, 1.58)	1.21 (0.87, 1.69)	1.23 (0.88, 1.74)	0.001	1.10 (0.78,1.55)	1.18 (0.84,1.68)	1.17 (0.83,1.65)	1.16 (0.82,1.67)	<0.001
HDL-cholesterol, mmol/L^b^	1.52 (0.35)	1.46 (0.34)	1.42 (0.34)	1.43 (0.36)	<0.001	1.54 (0.37)	1.46 (0.34)	1.43 (0.33)	1.39 (0.35)	<0.001
LDL-cholesterol, mmol/L^b^	2.94 (0.77)	2.99 (0.77)	3.04 (0.79)	3.07 (0.79)	<0.001	3.04 (0.79)	3.02 (0.79)	2.99 (0.77)	2.98 (0.78)	0.018
Total cholesterol, mmol/L^b^	5.18 (0.98)	5.19 (0.93)	5.21 (0.97)	5.27 (0.98)	0.010	5.34 (0.96)	5.26 (0.96)	5.15 (0.93)	5.11 (1.00)	<0.001
Fasting blood glucose, mmol/L^b^	5.50 (5.20, 6.00)	5.70 (5.30, 6.20)	5.80 (5.40, 6.50)	5.90 (5.40, 6.60)	<0.001	5.60 (5.30, 6.10)	5.70 (5.30, 6.20)	5.70 (5.40,6.30)	5.90 (5.40,6.60)	<0.001
Lymphocyte counts,10^9^/L^b^	1.83 (1.49, 2.24)	1.86 (1.52, 2.28)	1.87 (1.50, 2.32)	1.90 (1.50, 2.38)	0.003	2.03 (1.67, 2.46)	1.95 (1.60, 2.39)	1.84 (1.49, 2.28)	1.63 (1.30, 2.03)	<0.001
Neutrophil counts,10^9^/L^b^	3.00 (2.40, 3.74)	3.14 (2.53, 3.91)	3.34 (2.73, 4.07)	3.46 (2.76, 4.22)	<0.001	2.33 (1.96, 2.74)	2.99 (2.61, 3.43)	3.53 (3.06, 4.03)	4.40 (3.75, 5.15)	<0.001
Monocyte counts,10^9^/L^b^	0.37 (0.30, 0.45)	0.39 (0.32, 0.48)	0.41 (0.34, 0.51)	0.43 (0.35, 0.53)	<0.001	0.31 (0.26,0.37)	0.38 (0.32, 0.44)	0.43 (0.37, 0.50)	0.52 (0.43, 0.61)	<0.001
Diabetes mellitus, *N* (%)	260 (11.3)	371 (16.3)	507 (22.2)	540 (23.6)	<0.001	298 (13.0)	395 (17.3)	443 (19.4)	542 (23.7)	<0.001
Family history of stroke, *N* (%)	147 (6.5)	91 (4.0)	74 (3.3)	52 (2.3)	<0.001	105 (4.6)	93 (4.1)	82 (3.6)	88 (3.8)	0.357
Lipid-lowering drugs, *N* (%)	117 (5.1)	181 (8.0)	259 (11.3)	278 (12.2)	<0.001	186 (8.1)	207 (9.0)	216 (9.4)	226 (9.9)	0.204
Antihypertensive drugs, *N* (%)	205 (8.9)	443 (19.5)	718 (31.4)	883 (38.7)	<0.001	428 (18.7)	519 (22.7)	633 (27.7)	669 (29.2)	<0.001
Hypoglycemic agents, *N* (%)	102 (4.4)	157 (6.9)	202 (8.8)	238 (10.4)	<0.001	126 (5.5)	170 (7.4)	180 (7.9)	223 (9.7)	<0.001
Insulin, *N* (%)	36 (1.6)	39 (1.7)	54 (2.4)	51 (2.2)	0.145	24 (1.0)	39 (1.7)	49 (2.1)	68 (3.0)	<0.001
Aspirins, *N* (%)	150 (6.5)	194 (8.5)	259 (11.3)	285 (12.5)	<0.001	188 (8.2)	211 (9.2)	249 (10.9)	240 (10.5)	0.009
Thrombolytic agents, *N* (%)	13 (0.6)	11 (0.5)	23 (1.0)	14 (0.6)	0.134	13 (0.6)	13 (0.6)	17 (0.7)	18 (0.8)	0.713
Anticoagulant drugs, *N* (%)	21 (0.9)	18 (0.8)	37 (1.6)	38 (1.7)	0.008	21 (0.9)	33 (1.4)	29 (1.3)	31 (1.4)	0.399
Antibiotics, *N* (%)	198 (8.6)	167 (7.3)	167 (7.3)	147 (6.4)	0.049	181 (7.9)	171 (7.5)	159 (6.9)	168 (7.3)	0.668

SIRI, systemic inflammation response index; ePWV, estimated pulse wave velocity; SD, standard deviation; IQR, interquartile range.

Data were expressed as mean ± SD /median [interquartile ranges (IQR)] for continuous variables and percentage for categorical variables.

^a^ The normally distributed variable is presented as mean ± SD.

^b^ The non-normally distributed variable is presented as median (IQ).

Significant differences were observed in age, sex, educational level, BMI, drinking status, smoking status, SBP, DBP, fasting blood glucose, TC, TG, LDL-C, HDL-C, past medical history of DM and medication histories (including anti-hypertensive drugs, hypoglycemic agents, insulin, and aspirins) across the four quartiles of SIRI (*p* < 0.05) (Table 1). In Table 1, it was also observed that participants with higher ePWV levels had increased monocyte counts, lymphocyte counts, and neutrophil counts. On the other hand, individuals with higher SIRI levels exhibited elevated neutrophil counts and monocyte counts, but lower lymphocyte counts.

### 3.2. Association of each individual of ePWV and SIRI with incident stroke

**Figure 2:**
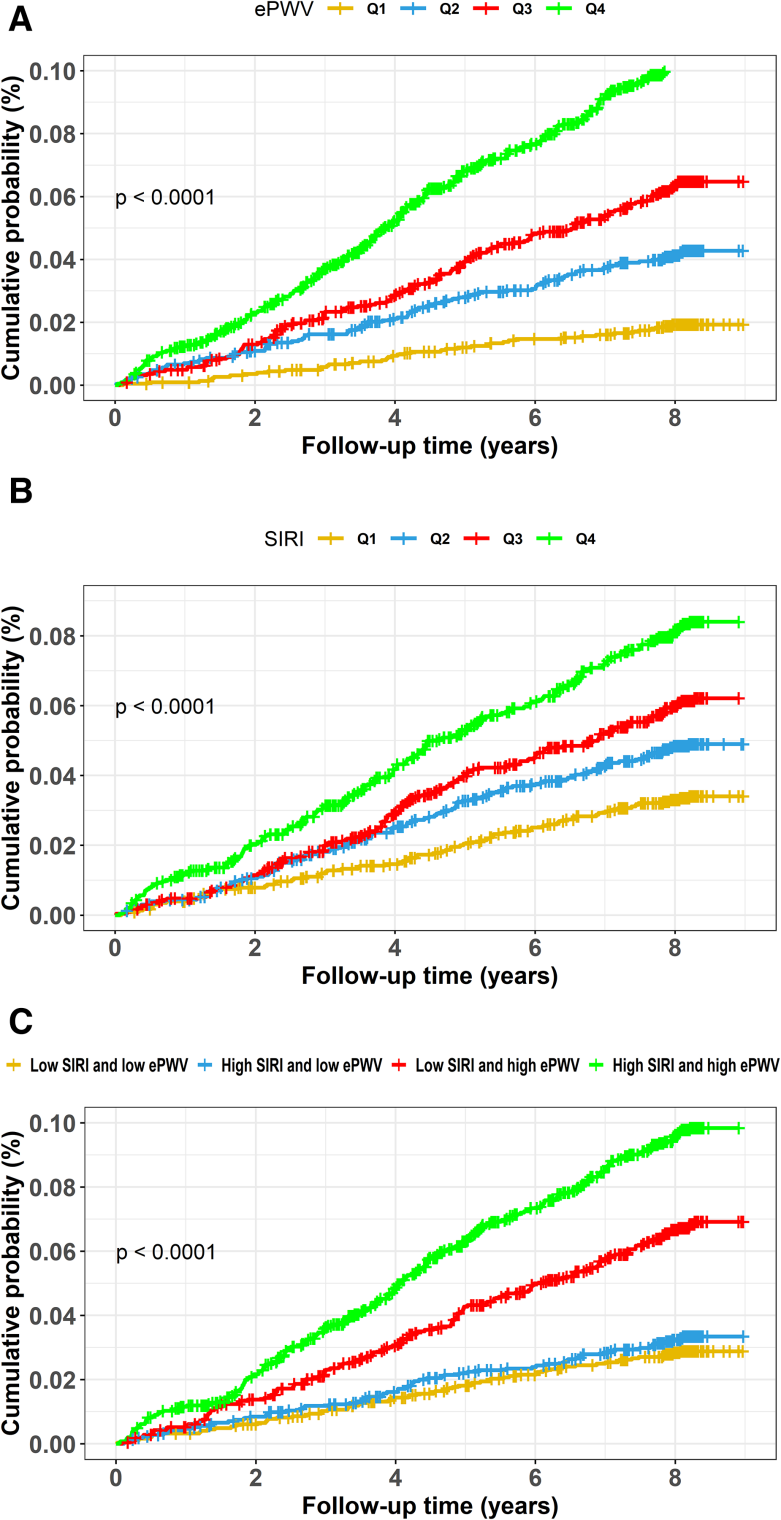
Cumulative hazard curves of total stroke for participants by quartiles of ePWV and SIRI and by combined statuses of SIRI and ePWV. Cumulative hazard curves of total stroke for participants by (**A**) quartiles of ePWV, (**B**) quartiles of SIRI, (**C**) combined statuses of SIRI and ePWV at high or low levels. ePWV, estimated pulse wave velocity; SIRI, systemic inflammation response index.

**Figure 3:**
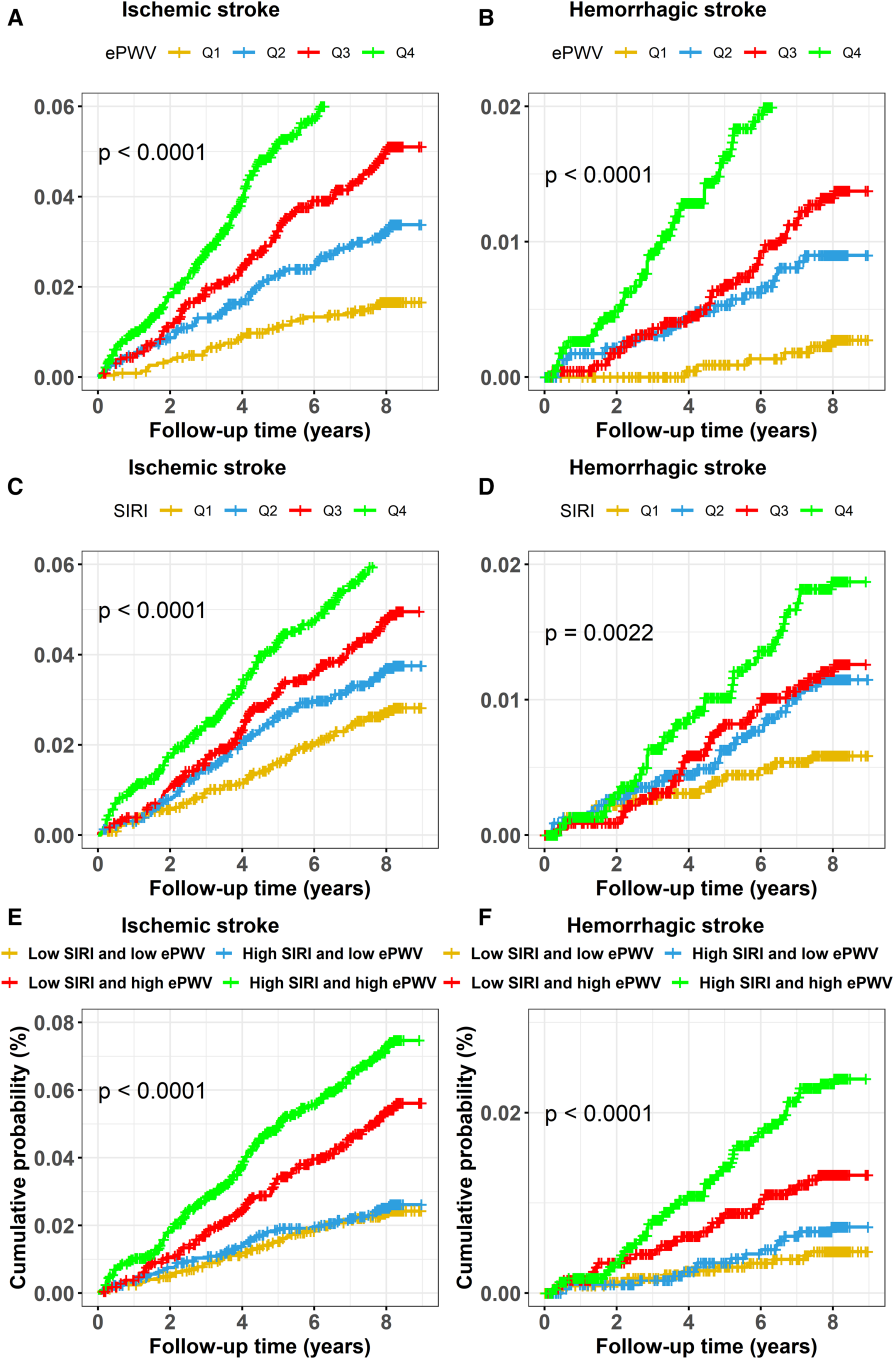
Cumulative hazard curves of stroke events for participants by quartiles of ePWV and SIRI and by combined statuses of ePWV and SIRI. Cumulative hazard curves of ischemic stroke for participants by (**A**) quartiles of ePWV, (**C**) quartiles of SIRI, (**E**) combined statuses of ePWV and SIRI at high or low levels, and cumulative hazard curves of hemorrhagic stroke for participants by (**B**) quartiles of ePWV, (**D**) quartiles of SIRI, (**F**) combined statuses of ePWV and SIRI at high or low levels. ePWV, estimated pulse wave velocity; SIRI, systemic inflammation response index.

Figures 2A, 3A,B exhibits the cumulative hazard curves across four quartiles of ePWV for stroke events (log rank test: *p* < 0.001 for all stroke events). The multivariate adjusted model showed that with each one-unit increase in the level of ePWV, the corresponding HRs (95% CI) for total stroke, ischemic stroke, and hemorrhagic stroke were 1.53 (95% CI, 1.23–1.90), 1.42 (95% CI, 1.11–1.83), and 1.92 (95% CI, 1.21–3.03), respectively (Table 2). Table 2 also shows the numbers, incidence rate and HRs (95% CI) of incident stroke and its subtypes among four quartiles of ePWV. In the fully adjusted Cox regression models, the highest quartiles of ePWV showed (suggestively) positive associations with total stroke (HR = 1.76, 95% CI, 0.98–3.16; *p* for trend = 0.081). Furthermore, when compared with the lowest quartiles of ePWV, the second and third quartile was separately associated with hemorrhagic stroke in the fully adjusted mode, with HRs (95% CI) being 3.71 (95% CI, 1.22–11.27) and 6.26 (95% CI, 1.59–24.69).

**Table 2:** Hazard ratios and 95% confidence intervals for incident stroke events by ePWV quartiles (*N* = 9,154).

Stroke events	ePWV	Event	Incidence rate(95% CI)^a^	Model 1^b^	Model 2^c^
Total stroke (*N* = 491)	1 m/s increase in ePWV			1.48 (1.35,1.63)	1.53 (1.23,1.90)
	Q1(≤8.84 m/s)	44	2.36 (1.75,3.17)	1.00 (ref)	1.00 (ref)
	Q2 (8.84–9.83 m/s)	96	5.32 (4.35,6.5)	1.90 (1.31,2.77)	1.35 (0.91,2.00)
	Q3 (9.83–10.96 m/s)	136	7.62 (6.44,9.01)	2.50 (1.7,3.68)	1.40 (0.89,2.20)
	Q4(≥10.96 m/s)	215	12.73 (11.14,14.56)	4.00 (2.58,6.22)	1.76 (0.98,3.16)
	*p* for trend^d^			<0.001	0.081
Ischemic stroke (*N* = 387)	1 m/s increase in ePWV			1.38 (1.23,1.53)	1.42 (1.11,1.83)
	Q1	38	2.04 (1.48,2.80)	1.00 (ref)	1.00(ref)
	Q2	77	4.27 (3.41,5.33)	1.6 (1.06,2.41)	1.15 (0.75,1.78)
	Q3	107	5.99 (4.96,7.24)	1.94 (1.27,2.96)	1.11 (0.68,1.82)
	Q4	165	9.77 (8.39,11.38)	2.74 (1.68,4.48)	1.27 (0.66,2.45)
	*p* for trend^d^			<0.001	0.518
Hemorrhagic stroke (*N* = 104)	1 m/s increase in ePWV			1.91 (1.58,2.31)	1.92 (1.21,3.03)
	Q1	6	0.32 (0.14,0.72)	1.00 (ref)	1.00 (ref)
	Q2	19	1.05 (0.67,1.65)	4.04 (1.55,10.51)	2.67 (0.98,7.28)
	Q3	29	1.62 (1.13,2.34)	7.03 (2.67,18.54)	3.71 (1.22,11.27)
	Q4	50	2.96 (2.24,3.91)	16.99 (5.94,48.6)	6.26 (1.59,24.69)
	*p* for trend^d^			<0.001	0.014

ePWV, estimated pulse wave velocity.

^a^ Incidence is defined as number of cardiovascular cases ⁄1,000 person-years.

^b^ Adjusted for baseline age and sex.

^c^ Adjusted for baseline age, sex, educational level, smoking status, drinking status, physical activity, body mass index, systolic blood pressure, anti-hypertensive drugs, total cholesterol, HDL- cholesterol, lipid-lowering drugs, diabetes mellitus, drug use for thrombus treatment, and family history of stroke.

^d^ Tests for linear trend were conducted by assigning median values of each quartile of ePWV as a continuous variable in the models.

Similarly, Figures 2B, 3C,D display the cumulative hazard curves across four quartiles of SIRI for stroke events (log rank test: *p* < 0.05 for all stroke events). With each one-unit increase in log-transformed levels of SIRI, the corresponding HRs (95% CI) for total stroke, ischemic stroke, and hemorrhagic stroke were 1.23 (95% CI, 1.04–1.47), 1.16 (95% CI, 0.96–1.41), and 1.52 (95% CI, 1.05–2.20), respectively (Table 3). Table 3 also shows the numbers, incidence rate and HRs (95% CI) of incident stroke and its subtypes among four quartiles of SIRI. In the fully adjusted Cox regression models in Table 3, the highest quartiles of SIRI showed positive associations with total stroke (HR = 1.55, 95% CI, 1.17–2.06; *p* for trend = 0.002), ischemic stroke (HR = 1.42, 95% CI, 1.04–1.94; *p* for trend = 0.018), and hemorrhagic stroke (HR = 2.17, 95% CI, 1.13–4.18; *p* for trend = 0.037), when compared with the lowest quartiles of SIRI.

**Table 3:** Hazard ratios and 95% confidence intervals for incident stroke events by SIRI quartiles (*N* = 9,154).

Stroke events	SIRI	Events	Incidence rate (95% CI)^a^	Model 1^b^	Model 2^c^
Total stroke (*N* = 491)	1-unit increase in log (SIRI)			1.36 (1.15,1.6)	1.23 (1.04,1.47)
	Q1(<0.48)	75	75,4.11 (3.28–5.15)	1.00 (ref)	1.00 (ref)
	Q2 (0.48–0.68)	107	107,5.93 (4.91–7.17)	1.3 (0.97,1.75)	1.23 (0.91,1.65)
	Q3 (0.68–0.99)	133	133,7.46 (6.3–8.85)	1.49 (1.12,1.98)	1.34 (1.01,1.79)
	Q4(≥0.99)	176	176,10.16 (8.76–11.78)	1.8 (1.36,2.37)	1.55 (1.17,2.06)
	*p* for trend^d^			<0.001	0.002
Ischemic stroke(*N* = 387)	1-unit increase in log (SIRI)			1.28 (1.06,1.55)	1.16 (0.96,1.41)
	Q1	62	62,3.4 (2.65–4.36)	1.00 (ref)	1.00 (ref)
	Q2	82	82,4.54 (3.66–5.64)	1.2 (0.86,1.67)	1.11 (0.8,1.55)
	Q3	106	106,5.95 (4.92–7.2)	1.42 (1.03,1.95)	1.28 (0.93,1.76)
	Q4	137	137,7.91 (6.69–9.35)	1.66 (1.22,2.26)	1.42 (1.04,1.94)
	*p* for trend^d^			0.001	0.018
Hemorrhagic stroke (*N* = 104)	1-unit increase in log (SIRI)			1.67 (1.17,2.41)	1.52 (1.05,2.2)
	Q1	13	0.71 (0.41–1.23)	1.00 (ref)	1.00 (ref)
	Q2	25	1.39 (0.94–2.05)	1.8 (0.92,3.53)	1.77 (0.9,3.46)
	Q3	27	1.52 (1.04–2.21)	1.83 (0.94,3.57)	1.66 (0.85,3.27)
	Q4	39	2.25 (1.64–3.08)	2.47 (1.3,4.71)	2.17 (1.13,4.18)
	*p* for trend^d^			0.009	0.037

SIRI, systemic inflammation response index.

^a^ Incidence is defined as number of cardiovascular cases ⁄1,000 person-years.

^b^ Adjusted for baseline age and sex.

^c^ Adjusted for baseline age, sex, educational level, smoking status, drinking status, physical activity, body mass index, systolic blood pressure, anti-hypertensive drugs, total cholesterol, HDL- cholesterol, lipid-lowering drugs, diabetes mellitus, drug use for thrombus treatment, and family history of stroke.

^d^ Tests for linear trend were conducted by assigning median values of each quartile of SIRI as a continuous variable in the models.

**Figure 4:**
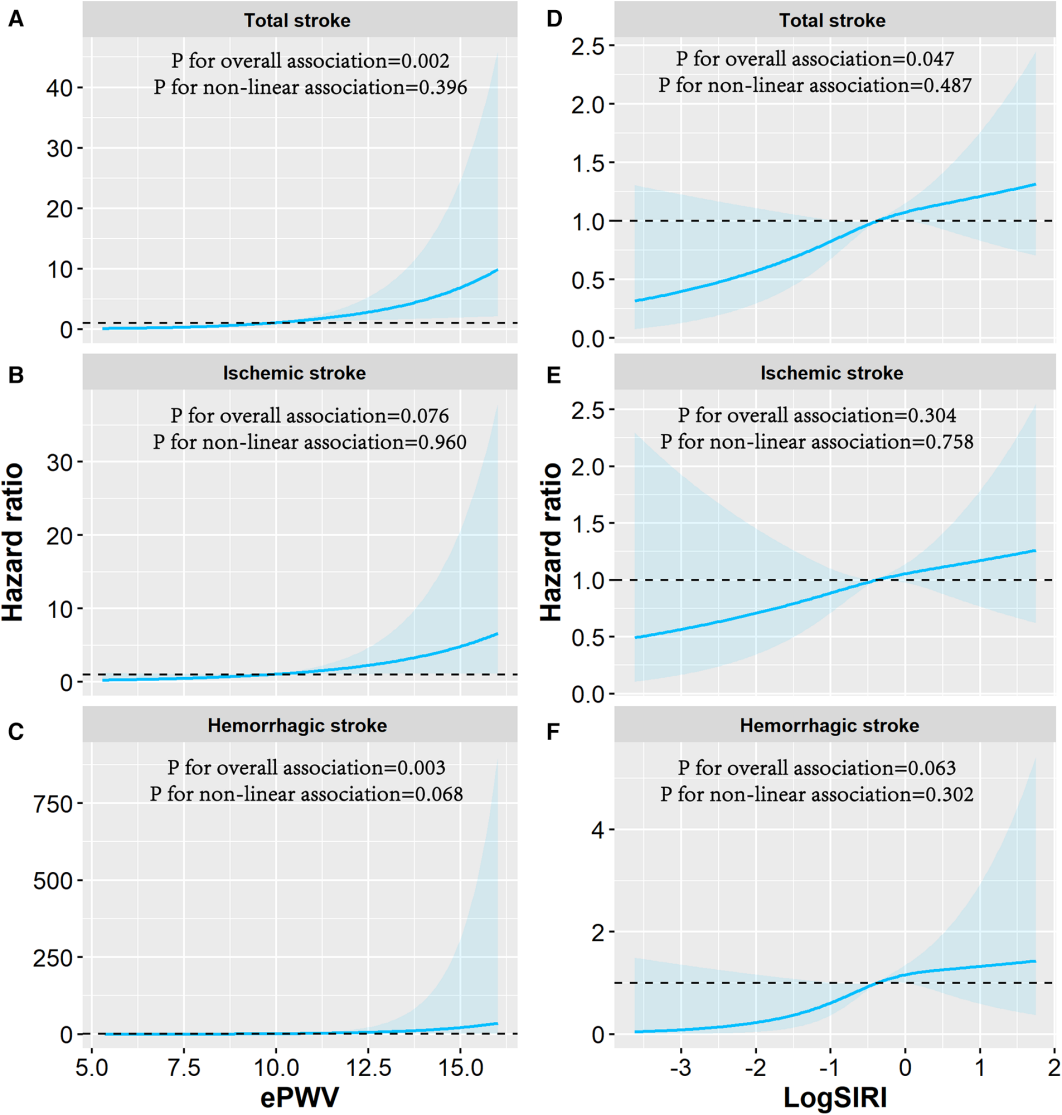
Dose-response association of log-transformed SIRI and ePWV with the risk of stroke events. Associations of ePWV with (**A**) total stroke, (**B**) ischemic stroke, (**C**) hemorrhagic stroke and associations of SIRI with (**D**) total stroke, (**E**) ischemic stroke, (**F**) hemorrhagic stroke were examined by multivariable Cox regression based on restricted cubic spline functions. The reference value and three knots in the spline were set to the median and the 5th, 50th, and 95th percentiles of the distribution of log-transformed ePWV and SIRI. Risk estimates were adjusted for baseline age, sex, educational level, smoking status, drinking status, physical activity, body mass index, hypertension, diabetes mellitus or hyperlipidemia, drug use for thrombus treatment, and family history of stroke. Solid line represents hazard ratios and shaded area represents the 95% CIs. ePWV, estimated pulse wave velocity; SIRI, systemic inflammation response index.

Figure 4 shows dose-response associations of ePWV and log-transformed SIRI with stroke events using restricted cubic splines. There were (suggestively) significant linear dose-response associations of ePWV with risks of total stroke (*p* for overall associations = 0.002; *p* for non-linear trend = 0.396), ischemic stroke (*p* for overall association = 0.076; *p* for non-linear trend = 0.960), and hemorrhagic stroke (*p* for overall associations = 0.003; *p* for non-linear trend = 0.068). In addition, there were (suggestively) significant linear dose-response associations of log-transformed SIRI with total stroke (*p* for overall associations = 0.047; *p* for non-linear trend = 0.487) and hemorrhagic stroke (*p* for overall associations =0.063; *p* for non-linear trend = 0.302).

### 3.3. Combined and interactive effects of ePWV and SIRI on incident stroke

Figures 2C, 3E,F exhibits the cumulative hazard curves of stroke events across the four groups categorized based on the combined status of ePWV and SIRI (log rank test: *p* < 0.001 for all stroke events). Table 4 shows combined effect of ePWV and SIRI at their low or high levels on stroke events**.** Compared to those with low levels of both ePWV and SIRI, participants with high levels of both ePWV and SIRI had increased risk of hemorrhagic stroke (multiple adjusted HR = 2.43, 95% CI, 1.09–5.42), but not total stroke (HR = 1.27, 95% CI, 0.90–1.79) and ischemic stroke (HR = 1.07, 95% CI, 0.73–1.57). However, a combined but not interactive effects between ePWV and SIRI on the risk of hemorrhagic stroke was observed (*p* for interaction = 0.647) in the multivariable adjusted model.

**Table 4:** Hazard ratios and 95% confidence intervals for incident stroke events by the status of SIRI and ePWV (*N* = 9,154).

Stroke events		Event	Incidence rate (95% CI)^a^	Model 1^b^	Model 2^c^
Total stroke (*N* = 491)	Low SIRI and low ePWV	71	3.60 (2.85,4.54)	1.00 (ref)	1.00 (ref)
	High SIRI and low ePWV	69	4.07 (3.22,5.16)	1.13 (0.81,1.58)	1.05 (0.76,1.47)
	Low SIRI and high ePWV	134	8.15 (6.88,9.65)	1.45 (1.05,2.00)	0.92 (0.64,1.31)
	High SIRI and high ePWV	217	11.83 (10.36,13.51)	2.16 (1.59,2.94)	1.27 (0.90,1.79)
Ischemic stroke(*N* = 387)	Low SIRI and low ePWV	60	3.04 (2.36,3.92)	1.00 (ref)	1.00 (ref)
	High SIRI and low ePWV	55	3.25 (2.49,4.23)	1.07 (0.74,1.54)	0.98 (0.68,1.42)
	Low SIRI and high ePWV	108	6.57 (5.44,7.93)	1.26 (0.88,1.80)	0.84 (0.56,1.24)
	High SIRI and high ePWV	164	8.94 (7.67,10.42)	1.76 (1.25,2.48)	1.07 (0.73,1.57)
Hemorrhagic stroke (*N* = 104)	Low SIRI and low ePWV	11	0.56 (0.31,1.01)	1.00 (ref)	1.00 (ref)
	High SIRI and low ePWV	14	0.83 (0.49,1.40)	1.47 (0.67,3.24)	1.44 (0.65,3.19)
	Low SIRI and high ePWV	26	1.58 (1.08,2.32)	2.57 (1.18,5.57)	1.36 (0.59,3.15)
	High SIRI and high ePWV	53	2.89 (2.21,3.78)	4.81 (2.33,9.94)	2.43(1.09,5.42)

SIRI, systemic inflammation response index; ePWV, estimated pulse wave velocity.

^a^ Incidence is defined as number of cardiovascular cases ⁄1,000 person-years.

^b^ Adjusted for baseline age and sex.

^c^ Adjusted for baseline age, sex, educational level, smoking status, drinking status, physical activity, body mass index, systolic blood pressure, anti-hypertensive drugs, total cholesterol, HDL- cholesterol, lipid-lowering drugs, diabetes mellitus, drug use for thrombus treatment, and family history of stroke.

### 3.4. Predicting efficiency of ePWV and SIRI on incident stroke events

Table 5 shows C-statistics for different models predicting total stroke, ischemic stroke and hemorrhagic stroke. The addition of ePWV (suggestively) significantly improved the Uno's concordance index beyond models with traditional cardiovascular risk factors for predicting total stroke from 0.684 (95% CI, 0.661–0.707) to 0.687 (95% CI, 0.664–0.710; *x*^2 ^= 6.65; *p* difference = 0.010), and for ischemic stroke from 0.684 (95% CI, 0.659–0.71) to 0.691(95% CI, 0.666–0.717; *x*^2 ^= 3.13, *p* difference = 0.077), respectively. Concomitantly adding the ePWV and SIRI into the conventional models produced similar predictive value.

**Table 5:** C-statistics for different models predicting incident stroke events.

Models		Concordance index (95% confidence interval)		
		Total stroke	Ischemic stroke	Hemorrhagic stroke
Model 1	Traditional risk factors	0.684 (0.661–0.707)	0.684 (0.659–0.71)	0.722 (0.674–0.771)
Model 2	SIRI + traditional risk factors	0.694 (0.671–0.716)	0.687 (0.661–0.712)	0.729 (0.681–0.776)
Model 3	ePWV + traditional risk factors	0.687 (0.664–0.710)	0.691 (0.666–0.717)	0.739 (0.696–0.783)
Model 4	ePWV + SIRI + traditional risk factors	0.696 (0.674–0.718)	0.693 (0.667–0.719)	0.744 (0.702–0.787)
Comparing uno's concordance statistic: model 2 vs. model 1		*x*^2^ = 1.81, *p* = 0.179	*x*^2^ = 0.87, *p *= 0.351	*x*^2^ = 1.65, *p *= 0.199
Comparing uno's concordance statistic: model 3 vs. model 1		*x*^2^ = 6.65, *p* = 0.010	*x*^2^ = 3.13, *p *= 0.077	*x*^2^ = 1.95, *p *= 0.163

SIRI, systemic inflammation response index; ePWV, estimated pulse wave velocity.

Traditional risk factors: baseline age, systolic blood pressure, anti-hypertensive drugs, diabetes mellitus, body mass index, current smoking status, total cholesterol, HDL-cholesterol, and family history of stroke.

### 3.5. Sensitivity analyses

When excluding participants with stroke events occurring during the first two years of follow-up, the main risk estimates were attenuated but were not statistically changed (Supplementary Tables S2–S4). When the main models were additionally adjusted for chronic bronchitis, asthma, and tuberculosis, similar findings were observed (Supplementary Tables S5–S7).

## 4. Discussion

This prospective cohort study investigated the associations of ePWV and SIRI with the risk of stroke in China. Our study found that both ePWV and SIRI were associated with an increased risk of total stroke and its subtypes. We also observed that individuals with elevated ePWV levels and high SIRI levels had a significantly higher risk of hemorrhagic stroke. Additionally, when ePWV was incorporated along with traditional cardiovascular risk factors, the predictive ability for total stroke and ischemic stroke (suggestively) were improved. These findings provide valuable insights for the development of more effective stroke prevention strategies.

Our results indicate that subjects with higher ePWV have a greater prevalence of established cardiovascular risk factors. Additionally, there were notable differences in educational level, smoking habits, alcohol consumption, and drug usage for cardiovascular disease prevention and treatment between the ePWV groups. These findings are consistent with previous studies that have reported a correlation between ePWV and established cardiovascular risk factors (35, 36). Vascular structure and BP are two crucial factors that influence arterial stiffness (13). With aging, the arterial wall gradually loses its elasticity, resulting in stiffening of the vessel wall and elevated risk of arterial stiffness. Additionally, arterial stiffness tends to increase with higher BP (13). Unhealthy lifestyle habits, including smoking, a high-fat diet, and a sedentary lifestyle, can also contribute to an increased risk of artery stiffness (37). Research has indicated that gender disparities in vascular stiffness are impacted by various factors such as age, obesity, hypertension, gender-specific risk factors, hormone status, diet, and exercise (38). Additionally, education plays a crucial role in determining one's socio-economic status (SES). Extensive evidence suggests that individuals with lower SES are more prone to risk factors like tobacco use and hypertension (39). Additionally, they tend to perceive stressors as posing a greater threat to their economic stability (40). These factors collectively contribute to the development of increased arterial stiffness.

In this study, a high level of ePWV was found to be associated with an increased risk of total stroke and its subtypes, and the association was particularly strong with hemorrhagic stroke. The findings provide further evidence for the link between stroke events and arterial stiffness measured by other indicators such as cfPWV (41) and baPWV (42). Our main findings were in line with a prospective study that followed middle-aged Caucasian men for a median of 28 years (12). Various speculative mechanisms have been suggested to explain the association between arterial stiffness and stroke events. Of these, it is important to highlight the hemodynamic changes that occur as a result of arterial stiffness. Prolonged elevation of pulse pressure can lead to arterial remodeling and thickening of the vessel wall, which thereby accelerates the development of plaque and atherosclerosis, ultimately resulting in the rupture or ulceration of the atherosclerotic plaque (13). Additionally, it has been observed that heightened arterial stiffness could potentially result in an excessive influx of flow pulsatility into cerebral small vascular beds (43). This, in turn, may induce haemodynamic stress, pulsatile pressure, or BP variability, ultimately leading to a “tsunami effect” on the cerebral parenchyma (43). These findings could potentially help elucidate why increased stiffness in the aorta is more likely to cause damage to microvessels and result in brain bleeding. Furthermore, our study revealed that ePWV (suggestively) has the ability to enhance the predictive value beyond that of traditional cardiovascular risk factors in determining the likelihood of total stroke and ischemic stroke. This provides supporting evidence for the improved prediction of stroke risk through additional assessment of aortic or carotid stiffness, surpassing the influence of other conventional risk factors (44, 45).

Our study found that a high level of SIRI was linked to an increased risk of total stroke events and its subtypes. Similarly, a separate study conducted in the Kailuan cohort in China also demonstrated consistent associations between SIRI and the occurrence of total stroke, as well as its subtypes (25). Notably, these associations were independent of CRP levels (25). These findings suggest that SIRI may provide insights into distinct biological aspects of the inflammatory response, different from those reflected by CRP. By incorporating information from monocyte, lymphocyte, and neutrophil counts, SIRI has the potential to reflect the three pathways involved in plaque formation, inflammatory response, and adaptive immune response (46). Previous studies have indicated that the recruitment of macrophages and T lymphocytes to plaques, the proliferation and polarization of monocyte/ macrophage, and neutrophil extracellular traps play important roles in the process of plaque proliferationand maturation, and cerebral thrombi (16, 46, 47). On the other hand, the mechanism behind systematic inflammation and hemorrhagic stroke is still not fully understood. However, it is known that hypertension aneurysm is a common cause of hemorrhagic stroke. One possible explanation is that the recruitment and infiltration of inflammatory cells can cause long-term changes in the vascular wall, leading to the development and progression of cerebral aneurysms (42).

Moreover, the presence of both high levels of ePWV and SIRI concomitantly further increased the risk of hemorrhagic stroke. These findings were a potential interpretation of the crosstalk between arterial stiffness and inflammation in the pathogenesis of stroke. Systemic inflammation, characterized by elevated levels of inflammatory markers/mediators in the bloodstream, can result in endothelial dysfunction (48), impairment of microvasomotor function (49), and reduced arterial elasticity (50). In individuals with high arterial stiffness, the pulsating energy generated by the heart's contraction is more strongly transmitted to the brain's microcirculation, potentially causing greater damage to blood vessels and contributing to vascular rupture (43, 51).

Inflammation plays a crucial role in the chronic process of atherosclerotic plaque formation and its complications in cerebral vessels. Therefore, targeting inflammation through anti-cytokine therapies is considered a promising therapeutic strategy to reduce the burden of cerebrovascular disease (52). Additionally, interventions aimed at reducing stiffness, such as pharmacological interventions or lifestyle modifications to influence BP, arterial function or arterial structure, have shown promise in reducing the prevalence of stroke (13). It is worth noting that the assessment of both ePWV and SIRI is readily available in clinical practice, and further studies are needed to determine whether the combined risk assessment of these factors can be used as an alternative target in trials to evaluate the effects of de-stiffening and anti-inflammatory therapies in reducing the risk of stroke in residents (53).

Our study had some strengths. This prospective study is the first to report on the individual and combined associations of ePWV and SIRI with stroke events, including ischemic stroke and hemorrhagic stroke, among middle-aged and older Chinese participants. SIRI, a composite marker comprising three different types of blood cells, may provide valuable insights into the interaction between various blood cells during the process of inflammation and immune response, surpassing the information obtained from a single blood cell type. Furthermore, the inclusion of ePWV has been found to enhance the predictive capacity beyond traditional stroke risk factors in determining the occurrence of stroke events. While these indicators cannot replace traditional risk assessment methods, they can serve as an additional tool to aid clinicians in assessing the incident risk of stroke events more effectively. Secondly, the combined stroke risk assessment of arterial stiffness and inflammatory markers, such as ePWV and SIRI, can be easily obtained from commonly assessed clinical parameters including age, BP, and routine blood tests. This easy access to the combined assessment may improve the non-invasive assessment of stroke risk (54). Additionally, these assessments could serve as a risk stratification tool for atherosclerosis or major cardiocerebrovascular events. They can provide a reference for identifying high-risk patients who may require prophylactic treatment or more regular medical examinations. Thirdly, this perspective study was conducted based on a large sample of middle-aged and elderly adults and collected a lot of information to control for most potential confounders.

There were some limitations of this study. First, SIRI was calculated from leukocyte counts of one-time routine blood test that may be influenced by specific pathological state, physiological conditions and some drugs usages of participants. To solve these issues, adding one more time measurements of blood tests would make the evaluation of chronic inflammation level more accurate over that of a single test (53). On the other hand, our analysis was conducted among participants without almost quite abnormal levels of inflammation, and adjusted for most covariates that could affect blood cell counts, such as alcohol consumption status, smoking status, BP and usages of drugs, to ensure the robustness of our results. The study was limited to a single sample of the cohort population, specifically middle-aged and older Chinese adults who had complete baseline blood pressure (BP) and blood test data. It is important to acknowledge that this group may only be a subset of the original cohort. Therefore, caution should be exercised when extrapolating the study's conclusions to the general population. Additionally, in our study, health-related data, including medical history, was assessed through a self-reported questionnaire. While this method may potentially affect the accuracy of the results, self-reported questionnaires have been widely used in population-based studies to obtain reliable estimates due to the significant cost associated with collecting objective health-related data for a large sample (45). Furthermore, the sample of participants with hemorrhagic stroke events occurring during the 7.22-year follow-up cohort were small and may influence the statistical power of analysis. This could potentially affect the statistical power of the analysis. Therefore, it is crucial to conduct larger prospective studies that include an adequate number of hemorrhagic stroke cases, which should specifically evaluate the association between ePWV and SIRI, as well as their combined effects, with each subtype of stroke. Last, C-reactive protein (CRP), a commonly used inflammatory marker, has been extensively investigated in the field of stroke risk assessment. Unfortunately, we were unable to collect CRP data during the initial survey, which prevented us from including it as a clinical parameter in our analysis. Given its importance, the absence of CRP data in our study may potentially impact affect the comprehensive analysis of stroke risk.

## 5. Conclusions

The presence of both high ePWV and SIRI individually, as well as together, was found to be associated with an increased risk of incident stroke. The indicator of ePWV could improve the predictive ability for total stroke and ischemic stroke.

## Data Availability

The datasets presented in this study can be found in online repositories. The names of the repository/repositories and accession number(s) can be found in the article/Supplementary Material.
