# Supplementary material for: Individual and combined associations of estimated pulse wave velocity and systemic inflammation response index with risk of stroke in middle-aged and older Chinese adults: a prospective cohort study
Source: Front Cardiovasc Med. 2023 Nov 3;10:1158098. doi: 10.3389/fcvm.2023.1158098 (PMC10655141; doi:10.3389/fcvm.2023.1158098)
Supplement: Supplementary file 1 [file Table1.docx]

**Table S1.** The baseline characteristics comparison between the included participants in the study and the stroke-followup cohort excluding cancer and cardiovascular diseases (CVD)

| Variables | The included participants | The stroke-followup cohort | p value |
| --- | --- | --- | --- |
| Participants (N) | 9154 | 19557 |  |
| Age, years, mean ± SD ^a^ | 63.03 (7.72) | 62.71 (7.64) | 0.001 |
| Female, N (%) | 4935 (53.9) | 11020 (56.3) | <0.001 |
| Education, N (%) |  |  | 0.002 |
| < 6 years | 2572 (28.1) | 5527 (28.5) |  |
| 6-8years | 3295 (36.0) | 7122 (36.7) |  |
| 9-11 years | 2226 (24.3) | 4779 (24.7) |  |
| ≥ 12 years | 1061 (11.6) | 1959 (10.1) |  |
| Body mass index, kg/m^2 b^ | 24.23 (3.31) | 24.34 (3.36) | 0.009 |
| Smoking status, N (%) |  |  | 0.344 |
| Current smokers | 1719 (18.8) | 3624 (18.7) |  |
| Ex-smokers | 946 (10.3) | 1905 (9.8) |  |
| Nonsmokers | 6488 (70.9) | 13891 (71.5) |  |
| Drinking status, N (%) |  |  | 0.351 |
| Current drinkers | 2020 (22.1) | 4349 (22.3) |  |
| Ex-drinkers | 436 (4.8) | 857 (4.4) |  |
| Nondrinkers | 6698 (73.2) | 14331 (73.4) |  |
| Systolic Blood pressure, mm Hg | 126.57 (17.97) | 129.30 (18.75) | <0.001 |
| Triglycerides, mmol/L ^b^ | 1.15 (0.82, 1.64） | 1.19 (0.85, 1.70） | <0.001 |
| HDL-cholesterol, mmol/L ^b^ | 1.46 (0.35) | 1.46 (0.41) | 0.869 |
| LDL-cholesterol, mmol/L ^b^ | 3.01 (0.78) | 3.03 (0.83) | 0.028 |
| Total cholesterol, mmol/L ^b^ | 5.22 (0.97) | 5.20 (0.96) | 0.247 |
| Lymphocyte counts,10^9^/L ^b^ | 1.86 (1.50, 2.31） | 1.91 (1.57, 2.35） | <0.001 |
| Neutrophil counts,10^9^/L ^b^ | 3.23 (2.59, 4.00） | 3.30 (2.65, 4.12） | <0.001 |
| Monocyte counts,10^9^/L ^b^ | 0.40 (0.33, 0.49） | 0.40 (0.33, 0.50） | 0.236 |
| Diabetes mellitus, N (%) | 1678 (18.3) | 3123 (17.8) | 0.309 |
| Family history of stroke, N (%) | 368 (4.0) | 882 (4.6) | 0.023 |
| Lipid-lowering drugs, N (%) | 835 (9.1) | 1589 (8.1) | 0.005 |
| Antihypertensive drugs, N (%) | 2249 (24.6) | 4767 (24.4) | 0.733 |
| Aspirins, N (%) | 888 (9.7) | 1701 (8.7) | 0.006 |
| Thrombolytic agents, N (%) | 61 (0.7) | 137 (0.7) | 0.803 |
| Anticoagulant drugs, N (%) | 114 (1.2) | 231 (1.2) | 0.684 |
| Antibiotics, N (%) | 679 (7.4) | 1414 (7.2) | 0.586 |

Abbreviations: SIRI, systemic inflammation response index; ePWV, estimated pulse wave velocity; SD, standard deviation; IQR, interquartile range.

Data were expressed as mean ± SD /median (interquartile ranges (IQR)) for continuous variables and percentage for categorical variables.

**Table S2.** Hazard ratios and 95% confidence intervals of ePWV for incident stroke events among participants excluding stroke cases in the first two years of follow-up (N=9,040)

| Stroke events | ePWV | Event | Incidence rate (95% CI ) ^a^ | Model 1^b^ | Model 2^c^ |
| --- | --- | --- | --- | --- | --- |
| Total stroke (N=377) | 1 m/s increase in ePWV |  |  | 1.48(1.32,1.64) | 1.52(1.18,1.95) |
|  | Q1(≤8.82 m/s) | 36 | 1.96(1.41,2.72) | 1.00 (ref) | 1.00 (ref) |
|  | Q2(8.82-9.82 m/s) | 70 | 3.84(3.04,4.85) | 1.58(1.03,2.41) | 1.09(0.69,1.69) |
|  | Q3(9.82-10.94 m/s) | 107 | 6.05(5.01,7.32) | 2.22(1.44,3.42) | 1.17(0.71,1.94) |
|  | Q4(≥10.94m/s) | 164 | 9.62(8.25,11.21) | 3.28(2.00,5.39) | 1.32(0.68,2.55) |
|  | p for trend^d^ |  |  | <0.001 | 0.383 |
|  | 1 m/s increase in ePWV |  |  | 1.38(1.22,1.56) | 1.41(1.05,1.88) |
| Ischemic stroke(N=293) | Q1 | 30 | 1.63(1.14,2.34) | 1.00 (ref) | 1.00 (ref) |
|  | Q2 | 56 | 3.07(2.36,3.99) | 1.35(0.85,2.16) | 0.93(0.57,1.53) |
|  | Q3 | 82 | 4.64(3.74,5.76) | 1.71(1.06,2.77) | 0.89(0.51,1.56) |
|  | Q4 | 125 | 7.33(6.15,8.74) | 2.26(1.29,3.96) | 0.91(0.43,1.91) |
|  | p for trend^d^ |  |  | 0.002 | 0.846 |
|  | 1 m/s increase in ePWV |  |  | 1.84(1.48,2.28) | 1.90(1.14,3.18) |
| Hemorrhagic stroke (N=84) | Q1 | 6 | 0.33(0.15,0.73) | 1.00 (ref) | 1.00 (ref) |
|  | Q2 | 14 | 0.77(0.45,1.30) | 2.80(1.03,7.63) | 1.93(0.67,5.58) |
|  | Q3 | 25 | 1.41(0.96,2.09) | 5.71(2.10,15.52) | 3.25(1.01,10.47) |
|  | Q4 | 39 | 2.29(1.67,3.13) | 11.90(3.94,35.92) | 4.98(1.13,21.94) |
|  | p for trend^d^ |  |  | <0.001 | 0.033 |

Abbreviations: ePWV, estimated pulse wave velocity.

^a^ Incidence is defined as number of cardiovascular cases ⁄1000 person-years.

^b^ Adjusted for baseline age and sex.

^c^ Adjusted for baseline age, sex, educational level, smoking status, drinking status, physical activity, body mass index, systolic blood pressure, anti-hypertensive drugs, total cholesterol, HDL-cholesterol, lipid-lowering drugs, diabetes mellitus, drug use for thrombus treatment, family history of stroke, chronic bronchitis, asthma, and tuberculosis.

^d^ Tests for linear trend were conducted by assigning median values of each quartile of ePWV as a continuous variable in the models.

**Table S3.** Hazard ratios and 95% confidence intervals of SIRI for incident stroke events among the participants excluding the cases in the first two years of follow-up (N=9,040)

| Stroke events | SIRI | Events | Incidence rate (95% CI) ^a^ | Model 1^b^ | Model 2^c^ |
| --- | --- | --- | --- | --- | --- |
|  | 1-unit increase in log(SIRI) |  |  | 1.29(1.07,1.56) | 1.18(0.97,1.43) |
| Total stroke (N=491) | Q1(<0.48) | 56 | 3.08(2.37,4.01) | 1.00 (ref) | 1.00 (ref) |
|  | Q2(0.48-0.68) | 84 | 4.67(3.77,5.78) | 1.36(0.97,1.91) | 1.281(0.91,1.8) |
|  | Q3(0.68-0.98) | 106 | 5.96(4.93,7.21) | 1.57(1.13,2.18) | 1.424(1.02,1.98) |
|  | Q4(≥0.98) | 131 | 7.52(6.34,8.93) | 1.75(1.27,2.42) | 1.527(1.1,2.12) |
|  | p for trend^d^ |  |  | 0.001 | 0.018 |
|  | 1-unit increase in log(SIRI) |  |  | 1.17(0.94,1.45) | 1.06(0.85,1.32) |
| Ischemic stroke(N=387) | Q1 | 49 | 2.7(2.04,3.57) | 1.00 (ref) | 1.00 (ref) |
|  | Q2 | 64 | 3.56(2.79,4.55) | 1.17(0.81,1.7) | 1.09(0.75,1.58) |
|  | Q3 | 81 | 4.55(3.66,5.66) | 1.34(0.94,1.92) | 1.21(0.84,1.74) |
|  | Q4 | 99 | 5.69(4.67,6.92) | 1.46(1.03,2.08) | 1.26(0.88,1.81) |
|  | p for trend^d^ |  |  | 0.030 | 0.186 |
| Hemorrhagic stroke (N=104) | 1-unit increase in log(SIRI) |  |  | 1.84(1.23,2.75) | 1.67(1.11,2.51) |
|  | Q1 | 7 | 0.39(0.18,0.81) | 1.00 (ref) | 1.00 (ref) |
|  | Q2 | 20 | 1.11(0.72,1.72) | 2.70(1.14,6.39) | 2.65(1.12,6.3) |
|  | Q3 | 25 | 1.41(0.95,2.08) | 3.19(1.37,7.44) | 2.93(1.25,6.85) |
|  | Q4 | 32 | 1.84(1.30,2.60) | 3.85(1.67,8.85) | 3.41(1.47,7.92) |
|  | p for trend^d^ |  |  | 0.003 | 0.013 |

Abbreviations: SIRI, systemic inflammation response index.

^a^ Incidence is defined as number of cardiovascular cases ⁄1000 person-years.

^b^ Adjusted for baseline age and sex.

^c^ Adjusted for baseline age, sex, educational level, smoking status, drinking status, physical activity, body mass index, systolic blood pressure, anti-hypertensive drugs, total cholesterol, HDL- cholesterol, diabetes mellitus, drug use for thrombus treatment, lipid-lowering drugs, and family history of stroke.

^d^Tests for linear trend were conducted by assigning median values of each quartile of SIRI as a continuous variable in the models.

**Table S4.** Hazard ratios and 95% confidence intervals of ePWV and SIRI for incident stroke among participants excluding stroke cases in the first two years of follow-up (N=9,040)

| Stroke events |  | Events | Incidence rate  (95% CI ) ^b^ | Model 1^b^ | Model 2^c^ |
| --- | --- | --- | --- | --- | --- |
| Total stroke | Low SIRI and low ePWV | 55 | 2.8(2.15,3.65) | 1.00 (ref) | 1.00 (ref) |
|  | High SIRI and low ePWV | 51 | 3(2.28,3.95) | 1.08(0.73,1.57) | 1.01(0.69,1.48) |
|  | Low SIRI and high ePWV | 106 | 6.46(5.34,7.82) | 1.46(1.01,2.11) | 0.93(0.62,1.39) |
|  | High SIRI and high ePWV | 165 | 9.01(7.73,10.49) | 2.11(1.49,2.99) | 1.25(0.85,1.85) |
| Ischemic stroke | Low SIRI and low ePWV | 47 | 2.39(1.8,3.19) | 1.00 (ref) | 1.00 (ref) |
|  | High SIRI and low ePWV | 39 | 2.3(1.68,3.14) | 0.96(0.63,1.47) | 0.89(0.58,1.36) |
|  | Low SIRI and high ePWV | 87 | 5.3(4.3,6.54) | 1.25(0.83,1.87) | 0.82(0.52,1.28) |
|  | High SIRI and high ePWV | 120 | 6.55(5.48,7.83) | 1.61(1.09,2.37) | 0.97(0.63,1.49) |
| Hemorrhagic stroke | Low SIRI and low ePWV | 8 | 0.41(0.2,0.81) | 1.00 (ref) | 1.00 (ref) |
|  | High SIRI and low ePWV | 12 | 0.71(0.4,1.24) | 1.72(0.7,4.21) | 1.71(0.7,4.19) |
|  | Low SIRI and high ePWV | 19 | 1.16(0.74,1.82) | 2.65(1.08,6.53) | 1.59(0.61,4.2) |
|  | High SIRI and high ePWV | 45 | 2.46(1.83,3.29) | 5.81(2.53,13.37) | 3.33(1.33,8.31) |

Abbreviations: SIRI, systemic inflammation response index; ePWV, estimated pulse wave velocity.

^a^ Incidence is defined as number of cardiovascular cases ⁄1000 person-years.

^b^ Adjusted for baseline age and sex.

^c^ Adjusted for baseline age, sex, educational level, smoking status, drinking status, physical activity, body mass index, systolic blood pressure, anti-hypertensive drugs, total cholesterol, HDL- cholesterol, lipid-lowering drugs, diabetes mellitus, drug use for thrombus treatment, and family history of stroke

| Stroke events | ePWV | Quartile of ePWV | | | | p for trend^b^ |
| --- | --- | --- | --- | --- | --- | --- |
|  |  | Q1 | Q2 | Q3 | Q4 |  |
|  |  | ≤8.84 | 8.84-9.83 | 9.83-10.96 | ≥10.96 |  |
| Total stroke | 1.53(1.23,1.9) | 1.00 (ref) | 1.351(0.91,2.01) | 1.402(0.89,2.2) | 1.76(0.98,3.17) | 0.08 |
| Ischemic stroke | 1.43(1.11,1.83) | 1.00 (ref) | 1.15(0.75,1.78) | 1.11(0.68,1.82) | 1.27(0.66,2.45) | 0.516 |
| Hemorrhagic stroke | 1.88(1.19,2.98) | 1.00 (ref) | 2.78(1.01,7.67) | 3.83(1.25,11.72) | 6.44(1.62,25.58) | 0.014 |

**Table S5.** Multivariable adjusted hazard ratios and 95% confidence intervals for incident stroke by ePWV quartiles (N=9,154) ^a^

Abbreviations: ePWV, estimated pulse wave velocity.

^a^Adjusted for baseline age, sex, educational level, smoking status, drinking status, physical activity, body mass index, systolic blood pressure, anti-hypertensive drugs, total cholesterol, HDL- cholesterol, lipid-lowering drugs, diabetes mellitus, drug use for thrombus treatment, family history of stroke, chronic bronchitis, asthma, and tuberculosis.

^b^ Tests for linear trend were conducted by assigning median values of each quartile of SIRI as a continuous variable in the models.

**Table S6.** Multivariable adjusted hazard ratios and 95% confidence intervals for incident stroke by SIRI quartiles (N=9,154) ^a^

| Stroke events | Log (SIRI) | Quartile of SIRI | | | |  |
| --- | --- | --- | --- | --- | --- | --- |
|  |  | Q1 | Q2 | Q3 | Q4 | p for trend^b^ |
|  |  | <0.48 | 0.48-0.68 | 0.68-0.99 | ≥0.99 |  |
| Total stroke | 1.23(1.03,1.46) | 1.00 (ref) | 1.227(0.91,1.65) | 1.348(1.01,1.8) | 1.543(1.16,2.05) | 0.003 |
| Ischemic stroke | 1.16(0.95,1.4) | 1.00 (ref) | 1.12(0.8,1.56) | 1.28(0.93,1.76) | 1.42(1.04,1.94) | 0.019 |
| Hemorrhagic stroke | 1.51(1.04,2.2) | 1.00 (ref) | 1.76(0.89,3.45) | 1.65(0.84,3.23) | 2.14(1.12,4.1) | 0.042 |

Abbreviations: SIRI, systemic inflammation response index.

^a^ Adjusted for baseline age, sex, educational level, smoking status, drinking status, physical activity, body mass index, systolic blood pressure, anti-hypertensive drugs, total cholesterol, HDL- cholesterol, lipid-lowering drugs, diabetes mellitus, drug use for thrombus treatment, family history of stroke, chronic bronchitis, asthma, and tuberculosis.

^b^ Tests for linear trend were conducted by assigning median values of each quartile of SIRI as a continuous variable in the models.

| Stroke events |  | Multiple adjusted HR (95% CI)^a^ |
| --- | --- | --- |
| Total stroke | Low SIRI and low ePWV | 1.00 (ref) |
|  | High SIRI and low ePWV | 1.04(0.75,1.46) |
|  | Low SIRI and high ePWV | 0.91(0.64,1.3) |
|  | High SIRI and high ePWV | 1.26(0.89,1.77) |
| Ischemic stroke | Low SIRI and low ePWV | 1.00 (ref) |
|  | High SIRI and low ePWV | 0.98(0.68,1.41) |
|  | Low SIRI and high ePWV | 0.84(0.56,1.24) |
|  | High SIRI and high ePWV | 1.07(0.73,1.57) |
| Hemorrhagic stroke | Low SIRI and low ePWV | 1.00 (ref) |
|  | High SIRI and low ePWV | 1.41(0.64,3.12) |
|  | Low SIRI and high ePWV | 1.33(0.57,3.09) |
|  | High SIRI and high ePWV | 2.38(1.07,5.3) |

**Table S7.** Multivariable adjusted hazard ratios and 95% confidence intervals for incident stroke by the status of SIRI and ePWV (N=9,154) ^a^

Abbreviations: SIRI, systemic inflammation response index; ePWV, estimated pulse wave velocity.

^a^ Adjusted for baseline age, sex, educational level, smoking status, drinking status, physical activity, body mass index, systolic blood pressure, anti-hypertensive drugs, total cholesterol, HDL- cholesterol, lipid-lowering drugs, diabetes mellitus, drug use for thrombus treatment, family history of stroke, chronic bronchitis, asthma, and tuberculosis.
