# Supplementary figures and images for: Individual and combined associations of estimated pulse wave velocity and systemic inflammation response index with risk of stroke in middle-aged and older Chinese adults: a prospective cohort study
Source: Front Cardiovasc Med. 2023 Nov 3;10:1158098. doi: 10.3389/fcvm.2023.1158098 (PMC10655141; doi:10.3389/fcvm.2023.1158098)

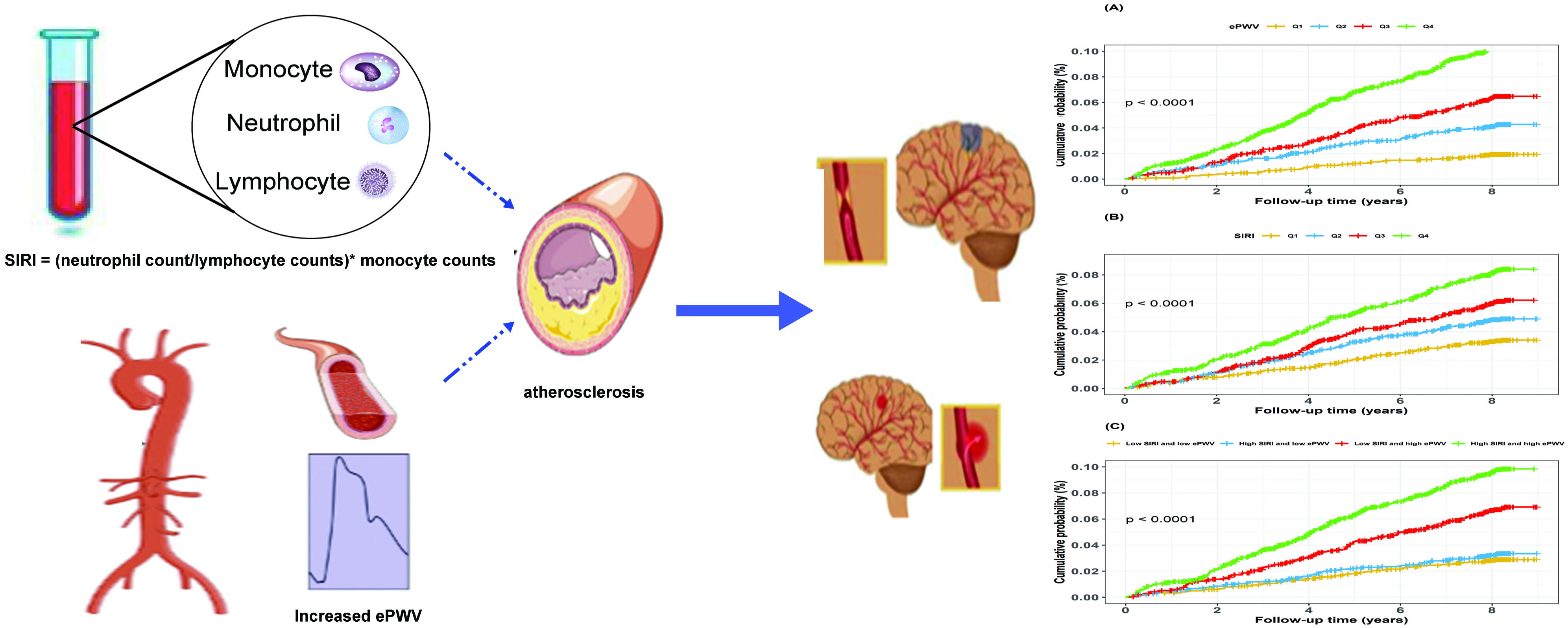

Supplement: Supplementary file 2 [file Image1.tif]
